# Postoperative radiotherapy in pIIIA-N2 non–small cell lung cancer after complete resection and adjuvant chemotherapy: A meta-analysis

**DOI:** 10.1097/MD.0000000000029550

**Published:** 2022-07-15

**Authors:** Shou-Feng Wang, Nai-Quan Mao, Wen-Hua Zhao, Xin-Bin Pan

**Affiliations:** a Department of Thoracic surgery, Guangxi Medical University Cancer Hospital, Nanning, Guangxi, People’s Republic of China; b Department of Respiratory Oncology, Guangxi Medical University Cancer Hospital, Nanning, Guangxi, People’s Republic of China; c Department of Radiation Oncology, Guangxi Medical University Cancer Hospital, Nanning, Guangxi, People’s Republic of China.

**Keywords:** chemotherapy, non–small cell lung cancer, NSCLC, pIII-N2, PORT, postoperative radiotherapy, surgery

## Abstract

**Background:**

This study aimed to evaluate the effect of postoperative radiotherapy (PORT) in patients with pIIIA-N2 non–small cell lung cancer after complete resection and adjuvant chemotherapy.

**Methods:**

Electronic databases (PubMed, Web of Science databases, Embase, and the Cochrane Central Register of Controlled Trials) were systematically searched to extract randomized control trials comparing PORT with observation in pIIIA-N2 non–small cell lung cancer patients until October 2021. Main outcomes were disease-free survival (DFS), overall survival (OS), and local recurrence.

**Results:**

Three-phase 3 randomized control trials involving 902 patients were included: 455 patients in the PORT group and 447 patients in the observation group. The methodological quality of the 3 randomized control trials were high quality. The pooled analysis revealed that PORT decreased local recurrence rate (odds ratio = 0.56, 95% confidence interval [CI]: 0.40–0.76). However, PORT did not improve median DFS (hazard ratio = 0.84, 95% CI: 0.71–1.00) and OS (hazard ratio = 1.02, 95% CI: 0.68–1.52).

**Conclusions:**

PORT decreased the incidence of local recurrence. However, PORT did not improve DFS and OS.

## 1. Introduction

Lung cancer is the leading cause of cancer death, which accounts for 18.0% of the total cancer deaths worldwide.^[1]^ The 5-year overall survival (OS) is as high as 53% after surgical resection in stage I-II non–small cell lung cancer (NSCLC).^[2]^ However, the 5-year OS of resected stage III NSCLC is <36%. The pIIIA-N2 is a risk factor of local recurrences and distant metastasis after complete resection.^[3]^

Adjuvant chemotherapy improves disease-free survival (DFS) and OS among patients with completely resected III NSCLC.^[4–6]^ Therefore, adjuvant chemotherapy is the standard treatment for patients with completely resected stage pIIIA-N2 NSCLC. On the other hand, patients with resected pIIIA-N2 NSCLC was believed to benefit from postoperative radiotherapy (PORT) that decreased the risk of local recurrences.^[7–11]^ Adjuvant chemotherapy followed by PORT was suggested to translate locoregional benefits from PORT and reduce distant metastasis into survival improvement. However, 2-phase 3 randomized clinical trials published recently suggested that PORT did not improve DFS and OS in patients with pIIIA-N2 NSCLC after complete resection and adjuvant chemotherapy.^[12,13]^ In order to identify the effect of PORT after adjuvant chemotherapy for resected stage III-N2 NSCLC patients, we performed this meta-analysis.

## 2. Methods

### 2.1. Data sources and searches

This study systematically searched the PubMed, Web of Science databases, Embase, and the Cochrane Central Register of Controlled Trials to search studies published until October 2021. The search process was based on the Preferred Reporting Items for Systematic Reviews and Meta-analyses reporting guidelines.^[14,15]^ The main search terms and their combinations included non-small cell lung cancer, NSCLC, postoperative radiotherapy, PORT, stage IIIA-N2, and randomized controlled trial. Relevant abstracts and presentations presented in major conference were also searched. Two researchers (S.-F.W. and .N.-Q.M) independently carried out the literature retrieval. If multiple articles covered the same study population, the study with the most recent and complete survival data was utilized. Any controversies were resolved by a third reviewer (X.-B.P.).

### 2.2. Study selection

Studies were included if they met the following criteria: randomized clinical phase 3 trials; patients with pIIIA-N2 NSCLC after complete resection and adjuvant chemotherapy; reporting data on disease-free survival (DFS), overall survival (OS), local–regional recurrence, distant metastases, or treatment-related adverse events (AEs) of grade 3 or higher for PORT group and observation group. Studies failing to meet these criteria were excluded.

### 2.3. Data extraction and quality assessment

Data extraction was performed by 2 authors (W.-H.Z. and X.-B.P.). Two authors (S.-F.W. and N.-Q.M.) separately assessed the methodological quality of included studies. The methodological quality of randomized clinical trial was assessed by a Cochrane risk of bias tool,^[16]^ which was consistent with the following 7 domains: random sequence generation; allocation concealment; blinding of participants and personnel; blinding of outcome assessment; incomplete outcome data; selective reporting; other bias. All disagreements were resolved in discussion, and consensus was reached.

### 2.4. Statistical analysis

The hazard ratio (HR) for survival outcomes (DFS and OS), the odds ratio (OR) for binary outcomes (local recurrence, distant metastasis, and treatment-related AEs), and their 95% confidence intervals (CIs) were used to measure outcomes and safety. An HR of <1 for DFS and OS was deemed preferable. An OR of <1 for local recurrence and distant metastasis was deemed preferable. An OR of >1 for treatment-related AEs grade 3 or higher indicated a greater likelihood of toxic effects.

*I*^2^ statistic was used to test statistical heterogeneity between studies. If there was no statistical heterogeneity (*I*^2^ <50%, *P* ≥ .1) among studies, fixed-effects model was used for OR and HR analysis. If there was statistical heterogeneity (*I*^2^ ≥50%, *P* < .1) among studies, random-effects model would be used. Forest plots were generated to show the estimated ORs and HRs, representing the theoretical gain in absolute percentage on the basis of endpoints. Upper limit and lower limit of 95% CIs were calculated.

All statistical analyses were performed using SPSS Statistics Version 26.0 software (IBM Co., Armonk, NY) and R software version 4.0.3 (http://www.R-project.org). *P* values were 2-tailed. *P* values of <.05 were considered statistically significant.

Ethical review and approval were waived for this study, due to all data deriving from public databases.

## 3. Results

### 3.1. Characteristics of included trials

**Figure 1.**
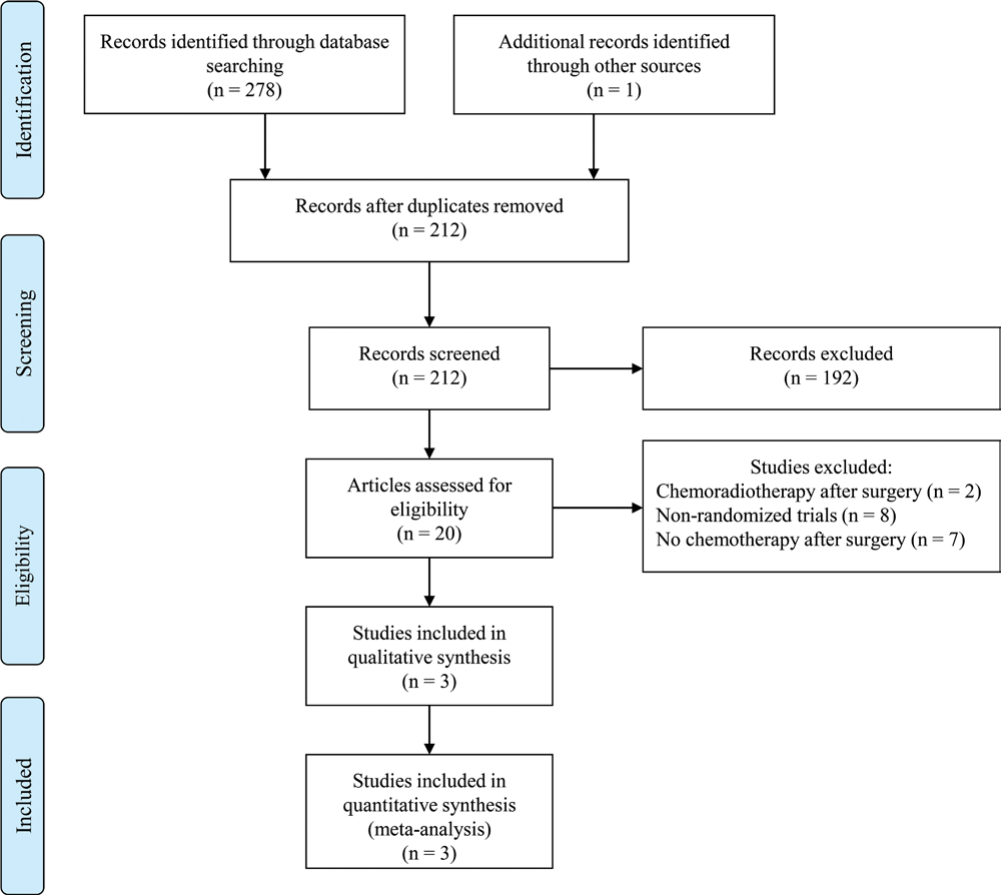
Flowchart depicting study selection.

**Figure 2.**
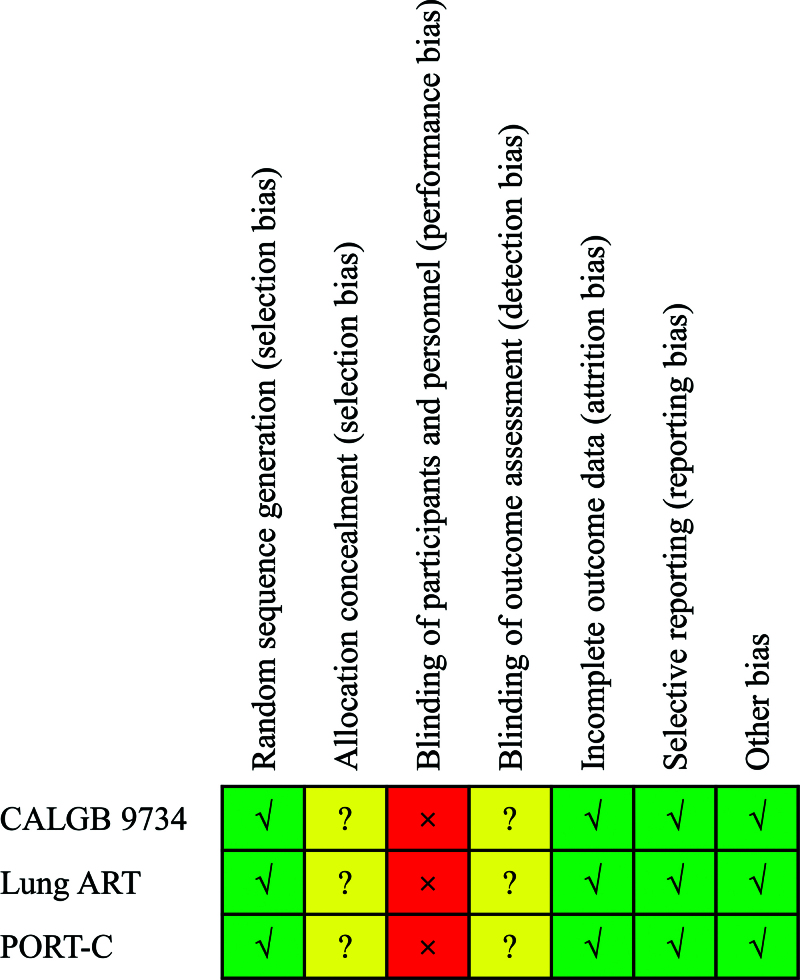
Risk of bias assessment of included studies. ART = adjuvant radiotherapy, CALGB = cancer and Leukemia Group B, PORT-C = postoperative radiotherapy-China.

Figure 1 shows the process of studies selection. This study screened 212 studies according to the primary search strategy. Figure 2 shows the methodological quality of included studies. Only 3 studies were included in our meta-analysis.^[12,13,17]^ The methodological quality of the 3 randomized control trials was high quality.

A total of 902 patients were included in this meta-analysis: 455 patients in the PORT group and 447 patients in the observation group. Table 1 summarizes the characteristics of all eligible studies. Postoperative radiotherapy-China (PORT-C) and Lung adjuvant radiotherapy (ART) trials used intensity-modulated radiotherapy or 3-dimensional conformal radiotherapy. PORT was administered 50 Gy in 25 fractions over 5 weeks in CALGB 9734 and PORT-C trials. In the Lung ART study, 54 Gy in 27 to 30 fractions was administered to patients in the PORT group. Patients received 4 cycles of platinum-based chemotherapy after surgery in CALGB 9734 and PORT-C trials. In the Lung ART trial, 12% patients in the observation group and 14% patients in the PORT group received neoadjuvant chemotherapy.

**Table 1:** Baseline characteristics of included studies.

Trials	Phase	Treatments	Participants	Median DFS (mo)	Median OS (mo)	3-yr DFS	3-yr OS	Local recurrence	Distant metastasis	Brain metastasis	Death due to cancer	Grade 3–5 AE
CALGB 9734	3	Observation	18	16.8	33.2	NR	NR	6	7	4	NR	NR
		PORT	19	33.7	41.5	NR	NR	5	6	1	NR	NR
Lung ART	3	Observation	249	22.8	NR	43.8%	68.5%	70	71	27	87/102	39
		PORT	252	30.5	NR	47.1%	66.5%	36	71	34	68/99	63
PORT-C	3	Observation	180	18.6	81.5	32.7%	82.8%	48	84	NR	42/47	0
		PORT	184	22.1	NR	40.5%	78.3%	39	91	NR	47/50	1

AE = adverse events, ART = adjuvant radiotherapy, CALGB = cancer and Leukemia Group B, DFS = disease-free survival, NR = not reported, OS = overall survival, PORT = postoperative radiotherapy, PORT-C = postoperative radiotherapy-China.

### 3.2. Effect of PORT on DFS

**Figure 3.**
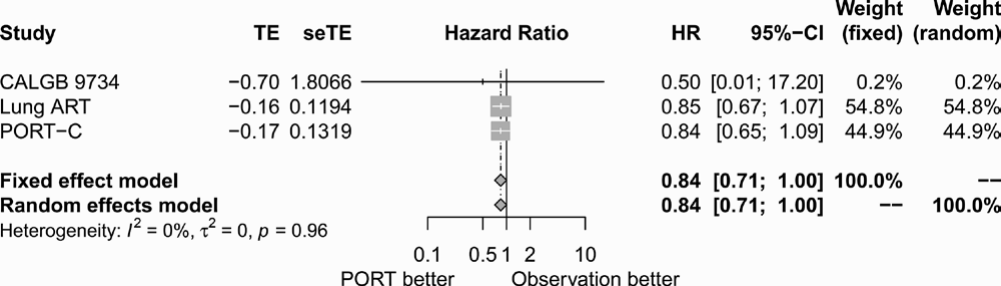
Forest plot of HR of median disease-free survival between postoperative radiotherapy and observation groups. ART = adjuvant radiotherapy, CALGB = cancer and Leukemia Group B, CI = confidence interval, HR = hazard ratio, PORT = postoperative radiotherapy, PORT-C = postoperative radiotherapy-China, seTE = standard error of treatment estimate, TE = treatment estimate.

Median DFS data were available from all the 3 trials. There was no significant heterogeneity among the 3 trials (*P* = .96, *I*^2^ = 0.00%). The fixed-effects model was used for meta-analysis. As shown in Figure 3, median DFS was not different between the PORT group and the observation group (HR = 0.84, 95% CI: 0.71–1.00).

### 3.3. Effect of PORT on OS

**Figure 4.**
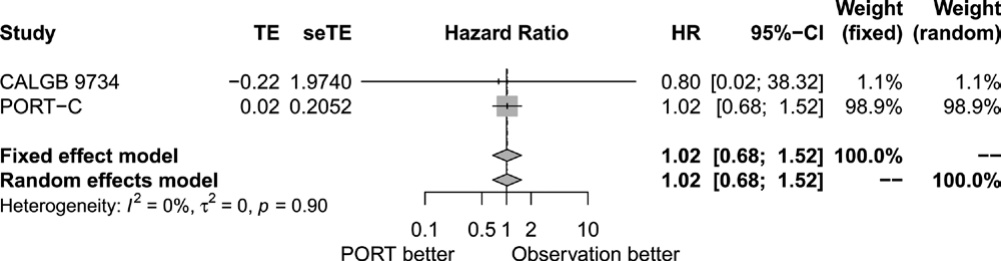
Forest plot of HR of median overall survival between postoperative radiotherapy and observation groups. CALGB = cancer and Leukemia Group B, CI = confidence interval, HR = hazard ratio, PORT = postoperative radiotherapy, PORT-C = postoperative radiotherapy-China, seTE = standard error of treatment estimate, TE = treatment estimate.

No significant heterogeneity among CALGB 9734 and PORT-C trials was observed (*P* = .90, *I*^2^ = 0.00%). The fixed-effects model was used for meta-analysis. Figure 4 shows that comparable median OS was found in the PORT group and the observation group (HR = 1.02, 95% CI: 0.68–1.52).

### 3.4. Effect of PORT on local recurrence

**Figure 5.**
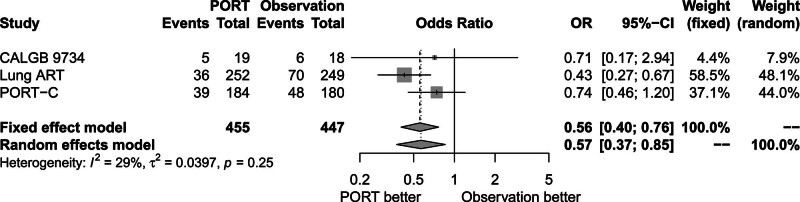
Forest plot of OR of local recurrence between postoperative radiotherapy and observation groups. ART = adjuvant radiotherapy, CALGB = cancer and Leukemia Group B, CI = confidence interval, OR = odds ratio, PORT = postoperative radiotherapy, PORT-C = postoperative radiotherapy-China.

There was no significant heterogeneity among the 3 trials (*P* = .25, *I*^2^ = 29.00%). The fixed-effects model was used for analysis. Figure 5 shows that local recurrence rate decreased in the PORT group compared to the observation group (OR = 0.56, 95% CI: 0.40–0.76).

### 3.5. Effect of PORT on distant metastases

**Figure 6.**
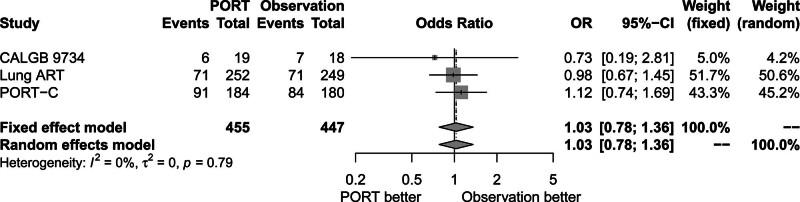
Forest plot of OR of distant metastasis between postoperative radiotherapy and observation groups. ART = adjuvant radiotherapy, CALGB = cancer and Leukemia Group B, CI = confidence interval, OR = odds ratio, PORT = postoperative radiotherapy, PORT-C = postoperative radiotherapy-China.

Figure 6 shows that distant metastases rate was similar between the PORT group and the observation group (OR = 1.03, 95% CI: 0.78–1.36). There was no significant heterogeneity among the 3 trials (*P* = .79, *I*^2^ = 0.00%). The fixed-effects model was used for analysis.

**Figure 7.**
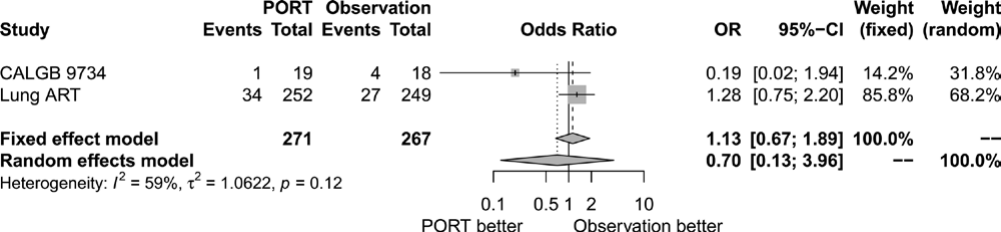
Forest plot of OR of brain metastasis between postoperative radiotherapy and observation groups. ART = adjuvant radiotherapy, CALGB = cancer and Leukemia Group B, CI = confidence interval, OR = odds ratio, PORT = postoperative radiotherapy.

Figure 7 shows that brain metastases rate was also comparable between the 2 groups (OR = 0.70, 95% CI: 0.13–3.96). There was heterogeneity between trials (*P* = .12, *I*^2^ = 59.00%). The random-effects model was used for analysis.

### 3.6. Effect of PORT on death due to cancer

**Figure 8.**
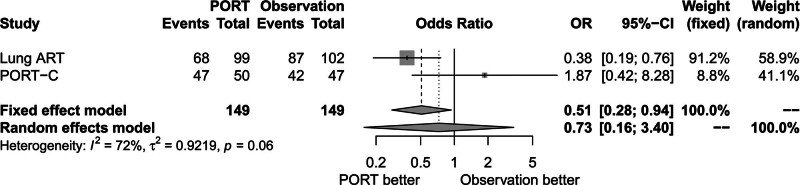
Forest plot of OR of death due to cancer between postoperative radiotherapy and observation groups. ART = adjuvant radiotherapy, CI = confidence interval, OR = odds ratio, PORT = postoperative radiotherapy, PORT-C = postoperative radiotherapy-China.

There was significant heterogeneity between trials (*P* = .06, *I*^2^ = 72.00%). The random-effects model was used for analysis. Figure 8 shows that death due to cancer rate was similar between the PORT group and the observation group (OR = 0.73, 95% CI: 0.16–3.40).

### 3.7. Effect of PORT on AE

**Figure 9.**
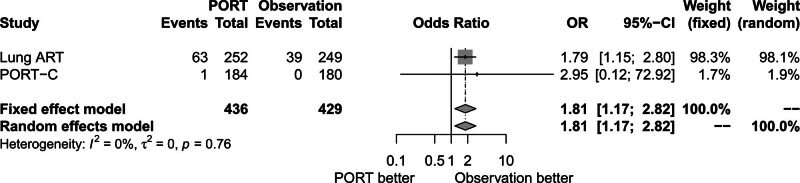
Forest plot of OR of treatment related adverse events of grade 3 or higher between postoperative radiotherapy and observation groups. ART = adjuvant radiotherapy, CI = confidence interval, OR = odds ratio, PORT = postoperative radiotherapy, PORT-C = postoperative radiotherapy-China.

Lung ART and PORT-C trials reported treatment-related AEs of grade 3 or higher. There was no significant heterogeneity between trials (*P* = .76, *I*^2^ = 0.00%). The fixed-effects model was used for analysis. Figure 9 shows that treatment-related AEs of grade 3 or higher rate increased in the PORT group compared to the observation group (OR = 1.81, 95% CI: 1.17–2.82).

## 4. Discussion

The role of PORT in pIIIA-N2 NSCLC is still unclear. Some retrospective cohort studies revealed that PORT improved OS.^[7–9]^ Moreover, several meta-analyses also demonstrated that PORT, with or without chemotherapy, significantly improved local control rates and DFS.^[10,11,18,19]^ However, PORT did not improve OS.^[19]^ Recently published Lung ART and PORT-C trials indicated that PORT did not improve DFS or OS.^[12,13]^ Thus, efficacy of PORT for resected pIIIA-N2 NSCLC needs to be reassessed.

Adjuvant chemotherapy is the standard treatment of patients with completely resected stage pIIIA-N2 NSCLC. Our meta-analysis included 3 randomized controlled trials with 902 patients to assess the effect of PORT in pIIIA-N2 NSCLC after complete resection and adjuvant chemotherapy. The results indicated that PORT improved the local control rates, while DFS and OS did not benefit from PORT. This study provided an updated, reliable, and comprehensive summary of effect of PORT in pIIIA-N2 NSCLC patients. The results provided reliable evidence for clinical practice and future research.

An individual participant data meta-analysis reported a significant adverse effect of PORT on survival, with an HR of 1.18, or an 18% relative increase in risk of death.^[20]^ This was equivalent to an absolute detriment of 5% (95% CI: 2%–9%) at 2 years, reducing OS from 58% to 53%. The deleterious effect of PORT might be attributed to an excess of intercurrent deaths, with a high incidence of cardiac and respiratory complications due to poor radiotherapy techniques.^[21,22]^ However, it was reported that radiation heart dosimetric parameters were not associated with OS.^[23]^ In support of this hypothesis, a meta-analysis was conducted based on modern PORT.^[11]^ The results reported modern PORT could decrease local recurrences and increase OS in patients with stage pIIIA-N2 NSCLC.

Patients included in our meta-analysis received intensity-modulated radiotherapy or 3-dimensional conformal radiotherapy. PORT with intensity-modulated radiotherapy or 3-dimensional conformal radiotherapy could guarantee sufficient irradiation doses to the target volume and decrease doses to organ at risk.^[24]^ However, the improvement of local–regional free survival did not translate into improvement of DFS or OS. The possible interpretations were the following: As pIIIA-N2 NSCLC is a heterogeneous group of diseases, some patients could benefit from PORT, but not all patients.^[25,26]^ Thus, further studies are needed to identify patients who will benefit from PORT using more detailed clinical features and molecular genetics information.^[27–29]^ Some patients might receive second-line or later therapies, but owing to limited data, their potential survival outcome benefits were not considered.

Limitations of this meta-analysis should be considered. First, this study could not perform subgroup analysis by stratifying patients by sex, smoking status, histology, the number of lymph nodes involved, or other factors that might be associated with the treatment outcomes due to the limited data on individual patients. These clinical characteristics should be assessed in future studies. Second, only 3 randomized controlled trials were included in this meta-analysis. Thus, the publication bias was not investigated. Third, sensitivity analysis was not performed due to the limited included studies. However, our study indicated that no significant changes were observed between fixed- effects model and random-effects model for pooled HRs and ORs. These results indicated that all the pooled results were stable and the overall tendency was consistent.

**Figure 10.**
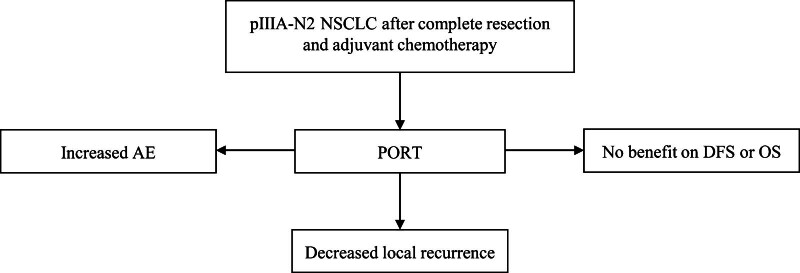
The final conclusion of this meta-analysis. AE = adverse event, DFS = disease-free survival, NSCLC = non–small cell lung cancer, OS = overall survival, PORT = postoperative radiotherapy.

The final conclusion of our study is shown in Figure 10. PORT decreased the incidence of local recurrence in patients with pIIIA-N2 NSCLC after complete resection and adjuvant chemotherapy. However, PORT did not improve DFS or OS. In the future, efficacy of PORT should be investigated combined with immune checkpoint inhibitors therapy and target therapy.^[30]^

## Author contributions

Conceptualization: Shou-Feng Wang, Xin-Bin Pan.

Data curation: Wen-Hua Zhao, Xin-Bin Pan.

Formal analysis: Shou-Feng Wang, Nai-Quan Mao.

Methodology: Shou-Feng Wang, Nai-Quan Mao.

Software: Xin-Bin Pan.

Validation: Shou-Feng Wang, Xin-Bin Pan.

Writing – original draft: Shou-Feng Wang, Nai-Quan Mao.

Writing – review & editing: Xin-Bin Pan.
